# Feasible Regions of Nozzle Temperature, Extrusion Pressure, and Printing Speed in Extrusion-Based Printing Using a Sodium Alginate–Carboxymethylcellulose–Collagen I Bioink

**DOI:** 10.3390/biomimetics11040281

**Published:** 2026-04-17

**Authors:** Evgenia Dimitriou, Nathan Wood, Hongmin Qin, Zhijian Pei

**Affiliations:** 1 Department of Industrial and Systems Engineering, Texas A&M University, College Station, TX 77843, USA; zjpei@tamu.edu; 2 Department of Biology, Texas A&M University, College Station, TX 77843, USA; woodn@tamu.edu (N.W.); hqin@bio.tamu.edu (H.Q.)

**Keywords:** additive manufacturing, material extrusion, bioprinting, bioink, feasible region

## Abstract

This study determines the feasible regions of nozzle temperature, extrusion pressure, and printing speed in extrusion-based printing using an acellular sodium alginate–carboxymethylcellulose–collagen I bioink. The tested range of nozzle temperature was from 10 to 35 °C in 5 °C increments, the range of printing speed was from 5 to 20 mm/s in 5 mm/s increments, and the range of extrusion pressure was from 10 to 100 kPa in 10 kPa increments. The feasible regions were defined as the combinations of process parameters that produced continuous extruded lines. Results show that continuous extruded lines were achieved at higher extrusion pressures (70–100 kPa) across most tested printing speeds and nozzle temperatures. In contrast, an extrusion pressure of 10 kPa resulted in discontinuous extruded lines under all tested combinations of nozzle temperature and printing speed, and an extrusion pressure of 20 kPa led to discontinuous extruded lines under all tested printing speeds and all tested temperatures except for 35 °C. Intermediate extrusion pressures required lower printing speeds to produce continuous extruded lines. These results highlight the interaction effects of extrusion pressure and printing speed on maintaining continuous extruded lines across the tested nozzle temperatures. These findings provide practical guidance for selecting extrusion pressures and printing speeds across different nozzle temperatures for printing of a sodium alginate–carboxymethylcellulose–collagen I bioink.

## 1. Introduction

The literature indicates a clear shift in drug discovery and toxicology from traditional animal models toward engineered tissues and organ-on-chip platforms [1,2]. Some of the engineered tissues and organ-on-chip platforms are fabricated using additive manufacturing (AM) [3,4]. AM refers to the layer-by-layer fabrication of three-dimensional structures directly from digital designs, while bioprinting represents a specialized subset of AM in which bioinks, often containing biomaterials and living cells, are deposited to create biologically relevant constructs [5]. It has been reported that bioprinted skin, lung, liver, cardiac, and tumor models are emerging in vitro platforms for preclinical screening, reproducing key structural and functional features of native tissues. It has been suggested that such platforms can partially replace animal experiments in specific applications, such as early-stage drug toxicity testing and disease modeling, by enabling testing on in vitro tissue models derived from human-relevant materials and cells [6,7,8,9,10].

Material extrusion (MEX) is an AM method that dispenses materials through a nozzle onto a printbed or the previously printed layer [11,12]. MEX has been employed with a wide variety of both cell-laden and acellular functional bioinks due to its ability to handle bioinks with high viscosities (up to 10^4^ Pa·s) [13]. It is crucial for the bioink to possess non-Newtonian, shear-thinning properties to reduce excessive fluid shear stress [14,15].

Depending on the mechanism that regulates extrusion, MEX bioprinters are classified as mechanical, solenoid or pneumatic [16]. Mechanical extrusion systems use a motor-driven piston or rotating screw to apply mechanical force to the bioink [17]. Solenoid-based extrusion systems rely on electromechanical valves that open and close rapidly to regulate bioink extrusion [18]. Pneumatic bioprinters use compressed air to extrude the bioink. Due to their relatively simple design, compatibility with a wide range of bioink viscosities, and ease of operation (e.g., adjustment of extrusion pressure), pneumatic bioprinters are particularly suitable for systematic investigation of printing parameters [19,20,21]. Extrusion pressure in pneumatic bioprinters can be controlled by varying the pressure of the compressed air. Bioink deposition can be further controlled by varying printing speed, which is the speed at which the printhead moves above the printbed, as well as nozzle temperature [22].

Table 1 summarizes process parameters, namely nozzle temperature, extrusion pressure, and printing speed, and their ranges used in reported studies on MEX-based bioprinting. Multicomponent bioinks based on sodium alginate and other natural polymers have been widely investigated. For example, alginate–collagen bioinks have been explored for tissue engineering applications because collagen facilitates cell adhesion while alginate contributes to extrusion quality [23,24]. Ternary or multipolymer bioinks have been investigated to balance printability and biological performance. For instance, bioinks incorporating alginate and carboxymethyl-based polysaccharides or other biopolymers have demonstrated improved rheological behavior and extrusion quality [25]. The incorporation of collagen also introduces temperature-dependent rheological behavior, since collagen undergoes thermal gelation that affects extrusion behavior [26]. Consequently, the interaction between bioink composition and printing parameters plays a critical role in determining extrusion quality. In parallel with experimental studies, data-driven approaches and machine-learning models have been used to identify relationships between rheological properties and printability, and accelerate optimization of bioink compositions and printing parameters for MEX-based bioprinting [27,28]. Together, these studies demonstrate the importance of both bioink nature and process parameters in determining extrusion quality. However, feasible regions of important process parameters that produce desired extrusion quality with multicomponent bioinks remain relatively unexplored.

Reported studies have investigated the printability of alginate-based bioinks and the effects of process parameters such as extrusion pressure, nozzle diameter and bioink composition on extrusion quality and dimensional fidelity [22,35,38,39,40]. However, these investigations have primarily focused on binary polymer bioinks or have examined the effects of individual process parameters in isolation. To date, no reported studies have established feasible regions of nozzle temperature, extrusion pressure, and printing speed in MEX-based bioprinting that consistently yield continuous extruded lines with a ternary, sodium alginate–carboxymethylcellulose–collagen I bioink. This paper will fill the gap in the literature by reporting an experimental study to determine the feasible regions of these parameters, using a ternary, sodium alginate–carboxymethylcellulose–collagen I bioink.

Thakare et al. (2020) reported their work to establish the feasible regions of bioink composition, extrusion pressure, and nozzle size for continuous MEX-based bioprinting. They used a bioink with different weight ratios of sodium alginate and methylcellulose [22]. Their work is different from the present study in two important aspects. First, the presence of collagen, a thermally responsive component, in the sodium alginate–carboxymethylcellulose–collagen I bioink of the present study introduces additional complexity to the extrusion process compared with the sodium alginate–methylcellulose bioink studied by Thakare et al., where thermal gelation effects were not present. Second, the present study identifies the feasible regions of nozzle temperature, extrusion pressure, and printing speed.

This paper has five sections. Following this section, Section 2 describes the materials and methods employed by this study and Section 3 provides the definition and assessment of feasible regions. Next, Section 4 presents the experimental results. Finally, Section 5 discusses the results, practical implications and limitations of the study, and proposes future research directions.

## 2. Materials and Methods

### 2.1. Materials

**Figure 1:**
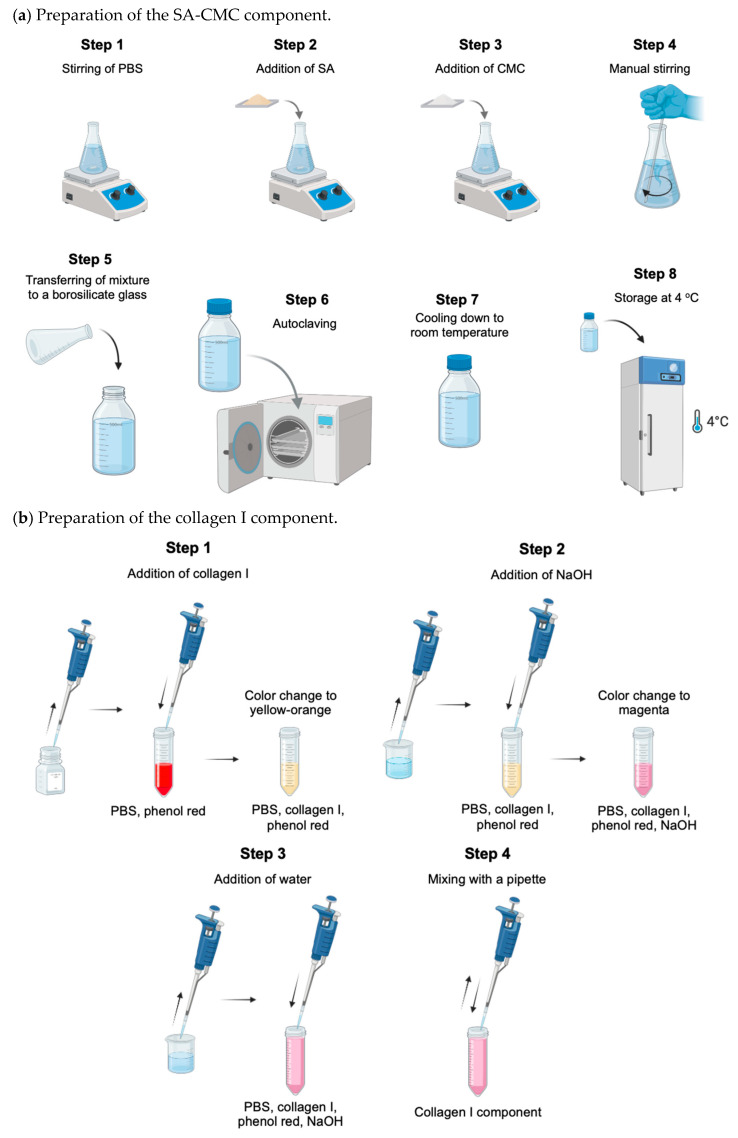

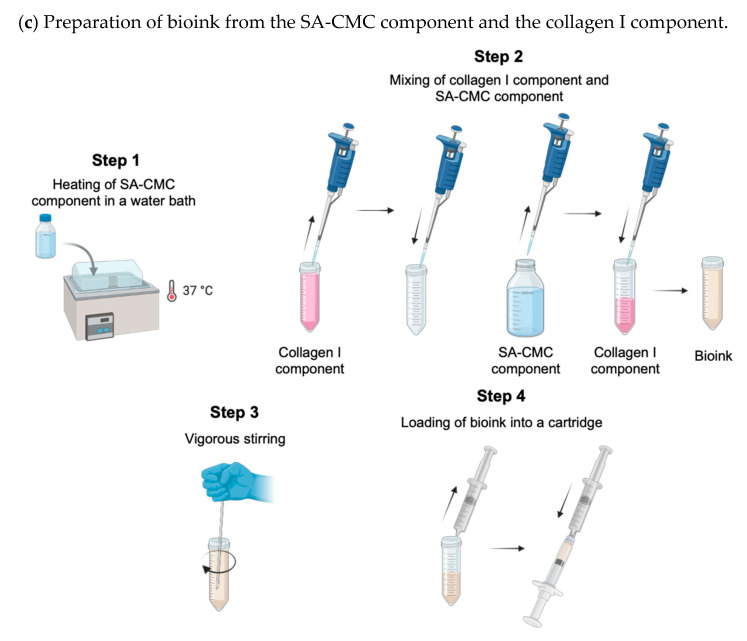
Steps of bioink preparation.

The bioink consisted of sodium alginate, SA (Sigma-Aldrich, Saint Louis, MO, USA; Cat. A112-250G, 4–12 cP as 1% *w*/*v* in water at 25 °C), carboxymethylcellulose, CMC (Thermo Fisher Scientific, Waltham, MA; CAS: 9004-32-4, 300–700 cP as 1% *w*/*v* in water at 25 °C), and collagen I (Telocol-10, Advanced Biomatrix, Carlsbad, CA, USA; with telopeptide regions). Two components were prepared for bioink synthesis, a SA (10% *w*/*v*)—CMC (4% *w*/*v*) component, and a collagen I component (8 mg·mL^−1^). The steps for the preparation of both components and the synthesis of the bioink are illustrated in Figure 1.

For the preparation of the SA-CMC component, illustrated in Figure 1a, 100 mL of phosphate-buffered saline (PBS, 1X), prepared according to the protocol described in [41], was stirred magnetically in a 200 mL Erlenmeyer flask at a temperature lower than 80 °C. Then, 10 g of SA was slowly added over the course of 1 h, and stirring continued until the SA was mostly dissolved. Next, 4 g of CMC was slowly added and dissolved via stirring. As the mixture became too viscous for magnetic stirring, manual stirring was then performed using an autoclaved glass stir rod for 1 min every 10–15 min, until no visible clumps were observed. Next, the mixture was transferred to a 500 mL borosilicate bottle and autoclaved on a 20 min sterilization cycle. No studies have been reported on the effects of autoclaving on combined SA-CMC. However, studies involving alginate–methylcellulose solutions have revealed that autoclaving reduces viscosity and compressive strength in a repeatable fashion [42]. This reduction in viscosity does not appear to significantly impact printability when compared to other sterilization methods, such as those using ultraviolet irradiation or supercritical carbon dioxide [42,43]. All SA-CMC batches were sterilized using the same autoclaving protocol; therefore, any potential effects of autoclaving were consistent across all experiments and were not expected to affect the identification of feasible regions. After autoclaving, the mixture was allowed to cool to room temperature and was stored at 4 °C and used within 1 day from the time of preparation to minimize potential changes in polymer properties prior to printing.

The collagen I component was prepared shortly before use, according to the steps illustrated in Figure 1b. First, 8 parts of collagen I solution were added to 1 part of PBS (10X) containing phenol red (Sigma-Aldrich; CAS: 143-74-8) (1 mM). Upon mixing collagen I with PBS, the phenol red turned yellow due to the acidity of the collagen I solution. Then, 0.25 parts of sodium hydroxide (NaOH, Sigma-Aldrich; CAS: 1310-73-2) solution (0.1 M) were added to the mixture, which was homogenized with a pipette. A color change was observed, from yellow to magenta. The remaining 0.75 parts of the collagen I component constituted autoclaved water.

For the preparation of the bioink, shown in Figure 1c, the SA-CMC component was heated to 37 °C in a water bath to improve flow and mixing when combined with the collagen I component. Next, it was mixed with the collagen I component in a 1:1 *v*/*v* ratio (bioink). The bioink was stirred vigorously for 2 min with an autoclaved rod. The bioink was loaded into a cartridge using a syringe. The final concentrations of SA, CMC and collagen I in the bioink were 5% *w*/*v*, 2% *w*/*v* and 4 mg·mL^−1^, respectively. These concentrations were selected based on bioink formulations with comparable compositions that have been previously reported by the authors of [44,45,46,47]. The final pH of the bioink was between 6.8 and 7.4. The bioink was stored at 4 °C until printing to avoid premature thermal gelation of collagen I before printing.

### 2.2. Characterization

Rheological analysis was performed using a rotational rheometer (Discovery Hybrid Rheometer DHR-2, TA Instruments, New Castle, DE, USA), with a Peltier plate allowing temperatures from room temperature to 200 °C, and a 40 mm parallel-plate geometry. The rheometer was connected to a cooler allowing temperatures from −40 °C to room temperature. The viscosity of the bioink and shear stress were measured at 10, 15, 20, 25, 30 and 35 °C. After loading onto the parallel-plate geometry, the bioink was allowed to equilibrate at the measurement temperature for 3 min prior to the start of the measurements. Viscosity and shear stress values were collected as a function of shear rate within a 10–10^5^ μN·m torque range, averaged from three replicate measurements. The measurement parameters were controlled using the TRIOS software package, version 6.0.0.726 (TA Instruments, New Castle, DE, USA). The zero-shear viscosity and yield stress of the bioink were determined via the Carreau model [48], and the Herschel–Bulkley model [49], respectively, using the TRIOS software package.

The rheological characterization was performed to provide a physical basis for the design and interpretation of the feasible region study. Since MEX-based bioprinting relies on pressure-driven flow through a nozzle, bioink viscosity directly affects the minimum extrusion pressure required to initiate flow, the stability of material deposition, and the sensitivity of extrusion behavior to printing speed and nozzle temperature.

### 2.3. Procedure and Conditions of Printing

The printing tests were performed on a pneumatic MEX-based bioprinter (BioX6, Cellink, Gothenburg, Sweden) equipped with a temperature-controlled printhead. A nozzle with a diameter of 0.2 mm was used. The process parameters selected for the study were nozzle temperature, extrusion pressure, and printing speed. Table 2 presents the values of these process parameters. The nozzle temperature was set between 10 and 35 °C in 5 °C increments. The nozzle temperature corresponded to the setpoint temperature of the temperature-controlled printhead. Although the nozzle and printbed temperatures could be controlled independently, printbed temperature was set at the same value as the nozzle temperature for all printing tests. The printbed temperature was intentionally matched to the nozzle temperature to eliminate thermal gradients between extrusion and deposition. This approach allowed the bioink to experience a consistent thermal history during flow and immediately after deposition, thereby mitigating the effect of temperature on bioink viscosity and ensuring better control of deposition. An upper limit of 35 °C was selected to avoid premature thermal gelation of collagen I during printing [45]. Printing at physiological temperature (37 °C) was therefore deferred to future studies involving cell-laden bioink. The printing substrate was a glass Petri dish. The Petri dish was placed on the printbed and allowed to equilibrate for 5 min to reach the printbed temperature. The bioink cartridge was inserted into the temperature-controlled printhead set to the desired temperature and allowed to equilibrate for 5 min for the bioink to reach the printhead temperature. Extrusion pressure was varied from 10 to 100 kPa in 10 kPa increments. The lower pressure limit (10 kPa) was determined through preliminary experiments as the minimum pressure required to initiate detectable bioink flow through the 0.2 mm nozzle. This value is consistent with previously reported extrusion pressures for bioinks of comparable formulation, printed through a nozzle with the same diameter [45]. Pressures above 100 kPa resulted in excessive flow and loss of extrusion control under the tested conditions (nozzle temperature and printing speed). The upper limit (100 kPa) was therefore selected to facilitate control of extrusion while remaining within the operating range of the bioprinter. Four values of printing speed were selected, 5, 10, 15 and 20 mm/s, based on values commonly reported in MEX-based bioprinting (Table 1). There were 240 unique combinations of process parameters. For each of these combinations, three replicates were printed. Prior to each print, extrusion pressure was briefly applied to allow a small amount of bioink to exit the nozzle, ensuring that the nozzle was fully filled with bioink, and that bioink could flow through the nozzle. The standoff distance between the nozzle and the substrate was 2 mm.

The replicates were evaluated immediately after deposition on the printbed. Therefore, no post-printing crosslinking or stabilization step was applied in this study. While sodium alginate typically requires ionic crosslinking (e.g., Ca^2+^) for long-term stabilization [46] and collagen I undergoes thermal gelation [45], these processes were outside the scope of the present work.

### 2.4. Design of Digital Model

Since the purpose of this study was to establish feasible regions of three process parameters before extending the analysis to more complex, application-specific geometries, three lines (three replicates) with a width of 0.2 mm and a length of 25 mm constituted the digital model for this study. More complex geometries would introduce additional effects such as directional changes, acceleration or deceleration effects, layer stacking, and path overlap, which could introduce variability and complicate the classification of the quality of the deposited structure. The digital model was designed using the DNA Studio 4 software integrated with the BioX6 3D printer. DNA Studio 4 offers a comprehensive interface that enables direct edits and generation of G-codes, thereby eliminating the need for external slicing software or manual file import.

## 3. Definition of Feasible Regions

**Figure 2:**
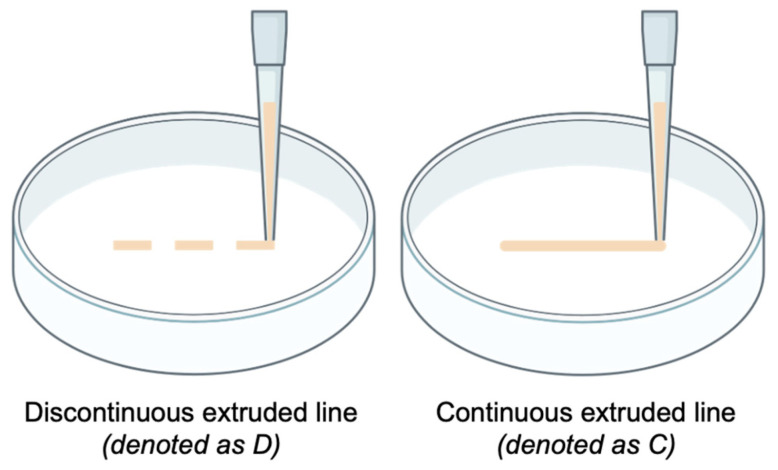
Two classifications of extruded lines.

Each extruded line (each replicate) was evaluated as follows: discontinuous extruded line (denoted as D) or continuous extruded line (denoted as C). An extruded line was considered as discontinuous when deposition was interrupted along the length of the line, whereas a continuous extruded line was obtained when deposition was uninterrupted along the entire length of the line. Figure 2 illustrates the two classifications for extruded lines.

For each combination of nozzle temperature, extrusion pressure and printing speed (240 combinations in total), three lines (three replicates) were extruded and evaluated as either discontinuous (D) or continuous (C). Therefore, there were four possible outcomes for each combination of process parameters: DDD, DDC, DCC, or CCC. Combinations in which all three extruded lines were discontinuous (DDD) were classified as “discontinuous extrusion”. Combinations in which at least one extruded line was discontinuous (DDC or DCC) were classified as “transient extrusion”. Conversely, combinations in which all three extruded lines were continuous (CCC) were classified as “continuous extrusion” (meaning that they were within the feasible regions). Replicates therefore aimed to confirm consistency for each parameter combination, enabling identification of feasible regions. This classification ensured that feasible regions corresponded to reproducible extrusion rather than isolated events. In other words, feasible regions were defined as the combinations of process parameters that yielded continuous extruded lines. Because the experiments were performed at discrete values of nozzle temperature, extrusion pressure, and printing speed, feasible regions represented ranges inferred from the tested parameter combinations rather than continuous ranges of process parameters. Similar approaches of classification have been used in reported studies on feasible regions in MEX-based bioprinting [22,50,51,52], although these studies employed numerical scoring systems instead of letters.

Since the objective of this study was to identify feasible regions of three printing parameters for continuous extrusion rather than to quantify dimensions of extruded lines, this two-level classification system was intentionally adopted to distinguish between discontinuous and continuous extrusion. Detailed dimension analysis (e.g., line width) will be addressed in a future study.

Additionally, because extruded lines were assessed using ordinal classification (discontinuous and continuous) rather than quantitative measurements, statistical analysis using means and standard deviations was not applicable.

## 4. Results

**Figure 3:**
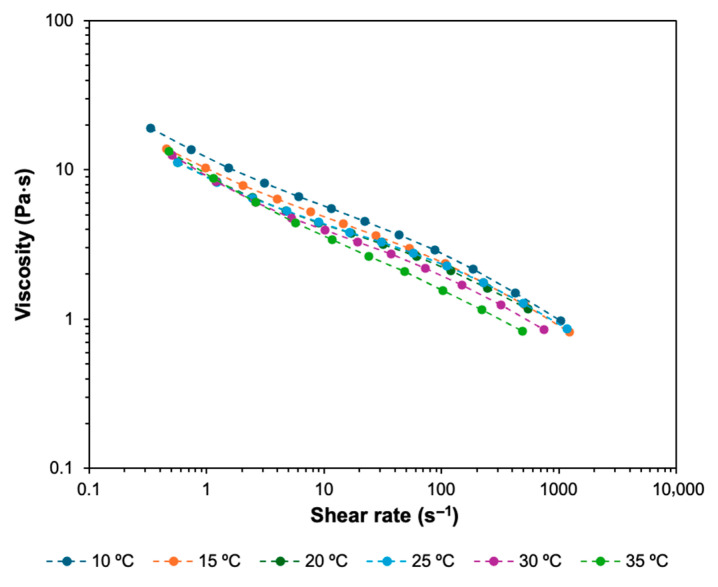
Viscosity of the bioink as a function of shear rate at different temperatures.

**Figure 4:**
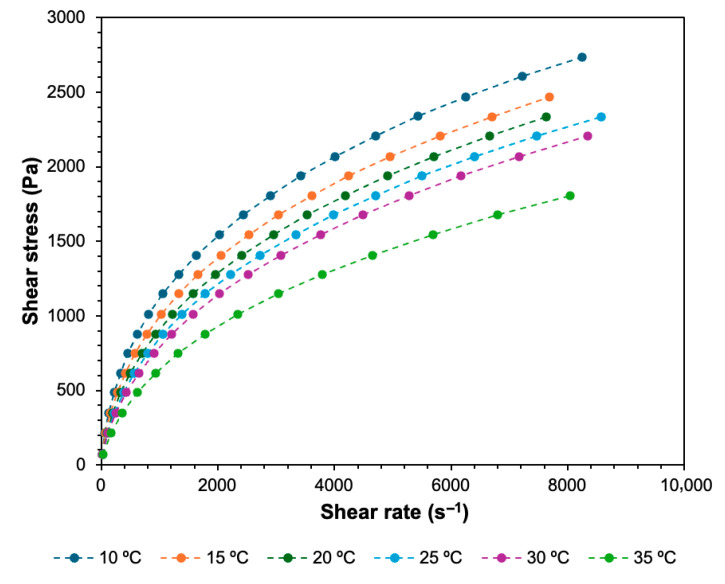
Shear stress as a function of shear rate at different temperatures.

Figure 3 and Figure 4 present the viscosity and shear stress measurements, respectively, performed with the bioink at 10, 15, 20, 25, 30 and 35 °C, as a function of shear rate. Table 3 summarizes the zero-shear viscosity data of the bioink at different temperatures, as well as the coefficient of determination, R^2^, for the Carreau model. The model provided an excellent fit to the experimental data, with R^2^ > 0.99 for all temperatures. Table 4 presents the yield stress data at different temperatures, and the coefficient of determination, R^2^, for the Herschel–Bulkley model. The Herschel–Bulkley model provided an excellent fit to the experimental data, with R^2^ > 0.99 across all tested temperatures.

The bioink exhibited strongly non-Newtonian and shear-thinning behavior at every temperature. This consistent behavior across all tested temperatures supported the homogeneity of the prepared bioink. Viscosity dropped by roughly two orders of magnitude across the shear rate range. The temperature increase from 10 to 30 °C reduced viscosity. This viscosity reduction was stronger at higher shear rates. In other words, the temperature increase intensified the shear-thinning behavior of the bioink. Despite the generally decreasing trend in zero-shear viscosity observed with increasing temperature, a slight increase in viscosity was recorded at 35 °C compared to 30 °C. This deviation was modest in magnitude and was attributed to the onset of the thermal gelation of collagen near physiological temperature, where fibril formation can increase structural organization within the collagen-containing bioink, thus increasing its low-shear viscosity [45,53,54]. Additionally, zero-shear viscosity values were obtained via Carreau model fitting, and small variations could have occurred from fitting sensitivity and experimental variation at low shear rates.

The relationship between shear stress and shear rate further supported the shear-thinning behavior or the bioink. Shear stress increased with increasing shear rate at all tested temperatures. However, this increase was non-linear, reflecting the non-Newtonian behavior of the bioink. Temperature also influenced the shear stress response of the bioink. Shear stress decreased as the temperature increased from 10 °C to 35 °C. This trend indicated that the resistance of the bioink to deformation (in this case shear) decreased with increasing temperature, which was consistent with the reduction in viscosity observed as temperature increased from 10 °C to 35 °C. The yield stress values obtained via the Herschel–Bulkley model decreased from 2.6218·10^2^ Pa at 10 °C to 1.1844·10^2^ Pa at 35 °C. Increasing temperature could increase polymer chain mobility and reduce intermolecular interactions, such as hydrogen bonding and chain entanglements, thereby decreasing the stress required to initiate flow [55,56]. At higher shear rates, the increase in shear stress became less intense, suggesting a structural rearrangement and alignment of polymer chains, which contributed to the shear-thinning behavior observed in both viscosity and shear stress results [35,57].

Such rheological behavior is advantageous for MEX-based bioprinting, as the bioink can maintain sufficient structural resistance at low shear rates and flow more easily under high shear rates encountered during extrusion through the nozzle.

Table 5 summarizes the classifications of the three extruded lines for each of the 240 combinations of nozzle temperature, extrusion pressure, and printing speed. Green cells represent combinations of continuous extrusion (CCC), corresponding to feasible regions, whereas orange cells represent combinations of discontinuous extrusion (DDD). Yellow cells represent combinations of transient extrusion (DDC or DCC).

Table 5a shows the classifications of the three extruded lines at different extrusion pressures and printing speeds for a nozzle temperature of 10 °C. Discontinuous extrusion was observed for extrusion pressures of 10 and 20 kPa at all tested printing speeds, and for extrusion pressures of 30, 40, 50 and 60 kPa at printing speeds of 10, 15 and 20 mm/s. Transient extrusion was observed at an extrusion speed of 50 kPa and a printing speed of 5 mm/s. Continuous extrusion, corresponding to feasible regions, was obtained for extrusion pressures of 30, 40 and 60 kPa at a printing speed of 5 mm/s, and for extrusion pressures of 70, 80, 90 and 100 kPa at all tested printing speeds.

The classifications of the extruded lines at different extrusion pressures and printing speeds for a nozzle temperature of 15 °C are shown in Table 5b. Extrusion was discontinuous for extrusion pressures of 10 and 20 kPa at all tested printing speeds. For extrusion pressures of 30 and 40 kPa, extrusion was discontinuous at printing speeds of 15 and 20 mm/s, transient at a printing speed of 10 mm/s, and continuous at a printing speed of 5 mm/s. For extrusion pressures of 50 and 60 kPa, discontinuous extrusion was observed at a printing speed of 20 mm/s, whereas printing speeds of 5, 10 and 15 mm/s led to continuous extrusion. Extrusion pressures of 70, 80, 90, and 100 kPa resulted in continuous extrusion at all tested printing speeds.

Table 5c presents the classifications obtained for a nozzle temperature of 20 °C. Discontinuous extrusion was observed for extrusion pressures of 10 and 20 kPa at all tested printing speeds. When extrusion pressure was 30 kPa, extrusion was discontinuous at printing speeds of 10, 15 and 20 mm/s, and transient at a printing speed of 5 mm/s. At 40 kPa, discontinuous extrusion was observed at a printing speed of 20 mm/s, transient extrusion was recorded at printing speeds of 10 and 15 mm/s, while extrusion was continuous at a printing speed of 5 mm/s. Extrusion pressures of 50 and 60 kPa resulted in transient extrusion at a printing speed of 20 mm/s, and in continuous extrusion at printing speeds of 5, 10, and 15 mm/s. Extrusion was continuous at higher extrusion pressures (60, 70, 80, 90 and 100 kPa) at all tested printing speeds.

The classifications of the extruded lines at different extrusion pressures and printing speeds for a nozzle temperature of 25 °C are shown in Table 5d. Discontinuous extrusion was observed at extrusion pressures of 10 and 20 kPa at all tested printing speeds. An extrusion pressure of 30 kPa led to discontinuous extrusion at printing speeds of 15 and 20 mm/s and transient extrusion at printing speeds of 5 and 10 mm/s. At 40 kPa, transient extrusion was observed at a printing speed of 20 mm/s, whereas extrusion was continuous at lower printing speeds. For extrusion pressures of 50 kPa and above, extrusion was continuous at all tested printing speeds.

Table 5e summarizes the classifications at different extrusion pressures and printing speeds for a nozzle temperature of 30 °C. Discontinuous extrusion occurred for extrusion pressures of 10 and 20 kPa at all tested printing speeds. For 30 and 40 kPa, discontinuous extrusion was observed at printing speeds of 10, 15 and 20 mm/s, while transient extrusion was recorded at a printing speed of 5 mm/s. An extrusion pressure of 50 kPa resulted in discontinuous extrusion when printing speed was 15 or 20 mm/s, and transient extrusion when printing speed was 5 or 10 mm/s. When extrusion pressure was 60 kPa, transient extrusion was observed at printing speeds of 10 and 20 mm/s, while at printing speeds of 5 and 15 mm/s, extrusion was continuous. Extrusion pressures of 70, 80 and 90 kPa resulted in continuous extrusion at all tested printing speeds, whereas extrusion at 100 kPa was continuous at a printing speed of 5 mm/s and discontinuous at higher printing speeds.

Finally, as shown in Table 5f for a nozzle temperature of 35 °C, extrusion was continuous at all tested combinations of extrusion pressure and printing speed, except when extrusion pressure was 10 kPa, which led to discontinuous extrusion at all tested printing speeds.

Finally, the trends observed in the feasible regions were consistent with the rheological behavior of the bioink. Lower viscosities at elevated temperatures reduced the extrusion pressure required for continuous extrusion but also increased sensitivity to printing speed. Similarly, the shear stress required to deform the bioink decreased with increasing temperature, reflecting the reduced resistance of the material to flow under shear stress. Consequently, the feasible regions identified in this study could be interpreted as combinations of nozzle temperature, extrusion pressure and printing speed that could overcome the bioink’s temperature-dependent resistance to flow while maintaining continuous extrusion.

The slight reduction in extrusion performance observed with the increase in temperature from 25 °C to 30 °C could reflect changes in the rheology of the SA–CMC–collagen bioink. Temperature variations could modify viscosity, shear stress response, and viscoelastic behavior, which may have affected extrusion quality under certain combinations of extrusion pressure and printing speed. Because the bioink consisted of multiple polymer components, the resulting temperature dependence of printing behavior was not necessarily monotonic. At 35 °C, collagen I may have begun to undergo thermal gelation, which could lead to time-dependent increases in viscosity [26,45]. The rheological measurements presented in this work showed a slight increase in viscosity at this temperature compared with lower temperatures, which reflected the onset of gelation of collagen I within the bioink. However, because the residence time of the bioink within the printhead prior to extrusion was limited, extensive collagen network formation was likely restricted.

## 5. Discussion and Future Research Directions

This experimental study determined the feasible regions of nozzle temperature, extrusion pressure and printing speed for bioprinting of a sodium alginate–carboxymethylcellulose–collagen I bioink. Feasible regions were defined as the ranges of these process parameters that resulted in continuous printed lines. Continuous extrusion was consistently achieved at higher extrusion pressures (70–100 kPa) across most tested nozzle temperatures and printing speeds. In contrast, an extrusion pressure of 10 kPa resulted in discontinuous extrusion under all tested combinations of nozzle temperature and printing speed, and an extrusion pressure of 20 kPa produced discontinuous lines under all tested printing speeds and temperatures except for 35 °C. Intermediate pressures required lower printing speeds for continuous extrusion.

The trends observed in this study are generally consistent with those reported in previous investigations of alginate-based bioinks. For example, they are consistent with the findings of Paxton et al. (2017) and Thakare et al. (2020), which showed that insufficient extrusion pressure and excessive printing speed can result in discontinuous extrusion [22,35].

By measuring viscosity and shear stress over the same temperature range as the nozzle temperature in the printing experiments, the rheological results clarified the role of temperature-dependent viscosity and yield stress, as well as shear-thinning behavior in governing the transition between discontinuous and continuous extrusion. The shear-thinning nature of the bioink facilitated extrusion through the narrow nozzle by reducing viscosity under the high shear conditions present during flow. At lower extrusion pressures, however, the applied stress may be insufficient to sustain continuous flow through the nozzle, resulting in discontinuous extrusion. Increasing the extrusion pressure increased the shear rate within the nozzle and enhanced extrusion quality. Printing speed also played an important role in extrusion quality: at higher speeds, the material could not be deposited at a rate sufficient to produce a continuous extruded line, particularly when combined with lower extrusion pressures. Temperature further affected these relationships by altering the viscosity of the bioink and therefore the extrusion pressure required to maintain extrusion.

The feasible regions identified in this study apply to the SA–CMC–collagen I bioink used, the specific nozzle diameter and the process parameters investigated, namely nozzle temperature, extrusion pressure, and printing speed. Changes in bioink composition, nozzle diameter or geometry, or printer configuration may alter the boundaries of these regions. Therefore, the results should be interpreted as feasible regions for the bioink and process parameters used here rather than as universally applicable printing conditions for all MEX-based bioprinting.

Beyond the specific bioink formulation investigated here, the approach used in this study for identifying feasible regions of process parameters may provide a useful framework for systematically mapping feasible regions in MEX-based bioprinting of other bioinks. Such feasible regions can help reduce trial-and-error in parameter selection and support more reliable fabrication of printed structures.

This study has several limitations that should be acknowledged. First, all experiments were conducted using a single nozzle diameter (0.2 mm). Variations in nozzle geometry may influence the boundaries of the feasible regions identified in this work. Additionally, the present study used a bioink formulation without cells. Its results should be validated in the future using cell-laden bioink, to ensure that the identified parameter ranges simultaneously maintain extrusion quality and cell viability. Finally, although the same autoclaving protocol was applied to bioink components to ensure consistency, future work should evaluate effects of autoclaving on bioink rheology and printability.

## Figures and Tables

**Table 1:** Summary of process parameters and their ranges in reported studies on MEX-based bioprinting.

Process Parameter	Reported Range	Reference
Nozzle temperature	15–35 °C	[29,30,31,32,33,34,35]
Extrusion pressure	70–500 kPa	[22,29,30,31,32,33,35]
Printing speed	1.67–25 mm/s	[36,37]

**Table 2:** Process parameters and their values.

Process Parameter	Value
Nozzle temperature (°C)	10, 15, 20, 25, 30, 35
Extrusion pressure (kPa)	10, 20, 30, 40, 50, 60, 70, 80, 90, 100
Printing speed (mm/s)	5, 10, 15, 20

**Table 3:** Zero-rate viscosity of the bioink at different temperatures.

Temperature (°C)	Viscosity (Pa·s)	Coefficient of Determination, R^2^
10	70.2546	0.997678
15	50.1153	0.997946
20	39.3789	0.996898
25	33.0885	0.996062
30	29.9029	0.997890
35	32.7314	0.999639

**Table 4:** Yield stress of the bioink at different temperatures.

Temperature (°C)	Yield Stress (Pa)	Coefficient of Determination, R^2^
10	2.6218·10^2^	0.999277
15	2.1966·10^2^	0.999385
20	1.9177·10^2^	0.999444
25	1.9187·10^2^	0.999432
30	1.5339·10^2^	0.999505
35	1.1844·10^2^	0.999558

**Table 5:** Feasible regions of nozzle temperature, extrusion pressure, and printing speed.

**(a) Nozzle Temperature: 10 °C**											
		**Extrusion Pressure (kPa)**									
		**10**	**20**	**30**	**40**	**50**	**60**	**70**	**80**	**90**	**100**
**Printing speed (mm/s)**	**5**	DDD	DDD	CCC	CCC	DCC	CCC	CCC	CCC	CCC	CCC
	**10**	DDD	DDD	DDD	DDD	DDD	DDD	CCC	CCC	CCC	CCC
	**15**	DDD	DDD	DDD	DDD	DDD	DDD	CCC	CCC	CCC	CCC
	**20**	DDD	DDD	DDD	DDD	DDD	DDD	CCC	CCC	CCC	CCC
**(b) Nozzle Temperature: 15 °C**											
		**Extrusion Pressure (kPa)**									
		**10**	**20**	**30**	**40**	**50**	**60**	**70**	**80**	**90**	**100**
**Printing speed (mm/s)**	**5**	DDD	DDD	CCC	CCC	CCC	CCC	CCC	CCC	CCC	CCC
	**10**	DDD	DDD	DDC	DCC	CCC	CCC	CCC	CCC	CCC	CCC
	**15**	DDD	DDD	DDD	DDD	CCC	CCC	CCC	CCC	CCC	CCC
	**20**	DDD	DDD	DDD	DDD	DDD	DDD	CCC	CCC	CCC	CCC
**(c) Nozzle Temperature: 20 °C**											
		**Extrusion Pressure (kPa)**									
		**10**	**20**	**30**	**40**	**50**	**60**	**70**	**80**	**90**	**100**
**Printing speed (mm/s)**	**5**	DDD	DDD	DCC	CCC	CCC	CCC	CCC	CCC	CCC	CCC
	**10**	DDD	DDD	DDD	DCC	CCC	CCC	CCC	CCC	CCC	CCC
	**15**	DDD	DDD	DDD	DDC	CCC	CCC	CCC	CCC	CCC	CCC
	**20**	DDD	DDD	DDD	DDD	DDC	DDC	CCC	CCC	CCC	CCC
**(d) Nozzle Temperature: 25 °C**											
		**Extrusion Pressure (kPa)**									
		**10**	**20**	**30**	**40**	**50**	**60**	**70**	**80**	**90**	**100**
**Printing speed (mm/s)**	**5**	DDD	DDD	DDC	CCC	CCC	CCC	CCC	CCC	CCC	CCC
	**10**	DDD	DDD	DDC	CCC	CCC	CCC	CCC	CCC	CCC	CCC
	**15**	DDD	DDD	DDD	CCC	CCC	CCC	CCC	CCC	CCC	CCC
	**20**	DDD	DDD	DDD	DDC	CCC	CCC	CCC	CCC	CCC	CCC
**(e) Nozzle Temperature: 30 °C**											
		**Extrusion Pressure (kPa)**									
		**10**	**20**	**30**	**40**	**50**	**60**	**70**	**80**	**90**	**100**
**Printing speed (mm/s)**	**5**	DDD	DDD	DDC	DDC	DCC	CCC	CCC	CCC	CCC	CCC
	**10**	DDD	DDD	DDD	DDD	DDC	DCC	CCC	CCC	CCC	DDD
	**15**	DDD	DDD	DDD	DDD	DDD	CCC	CCC	CCC	CCC	DDD
	**20**	DDD	DDD	DDD	DDD	DDD	DCC	CCC	CCC	CCC	DDD
**(f) Nozzle Temperature: 35 °C**											
		**Extrusion Pressure (kPa)**									
		**10**	**20**	**30**	**40**	**50**	**60**	**70**	**80**	**90**	**100**
**Printing speed (mm/s)**	**5**	DDD	CCC	CCC	CCC	CCC	CCC	CCC	CCC	CCC	CCC
	**10**	DDD	CCC	CCC	CCC	CCC	CCC	CCC	CCC	CCC	CCC
	**15**	DDD	CCC	CCC	CCC	CCC	CCC	CCC	CCC	CCC	CCC
	**20**	DDD	CCC	CCC	CCC	CCC	CCC	CCC	CCC	CCC	CCC
**Combinations of discontinuous extrusion**				**Combinations of** **transient extrusion**				**Combinations of** **continuous extrusion** **(within feasible regions)**			

## Data Availability

The original contributions presented in this study are included in the article. Further inquiries can be directed to the corresponding author.
